# Characterization and diversity of rhizobia nodulating selected tree legumes in Ghana

**DOI:** 10.1007/s13199-016-0383-1

**Published:** 2016-02-18

**Authors:** Emmanuel Yaw Boakye, Innocent Yao Dotse Lawson, Seth Kofi Akyea Danso, Samuel Kwame offei

**Affiliations:** College of Agriculture and Consumer Sciences, Department of Soil Science, University of Ghana Legon, P. O. Box 245, Legon, Ghana

**Keywords:** Characterization, Diversity, Rhizobium, Tree legumes

## Abstract

The study was conducted to assess the characteristics and diversity of the rhizobia that nodulate some prominent tree legumes in three soils of Ghana. Five introduced and/or indigenous tree legumes were initially assessed for nodulation in three Ghanaian soils. After 12 weeks of growth in nursery pots the 200 rhizobial strains isolated from their nodules were characterized culturally, metabolically and phenotypically. Sixty of these isolates were selected randomly and their genotypic characteristics determined using PCR-RFLP of 16S rRNA and intergenic spacer (ITS) genes. Each tree legume was nodulated by isolates classified as fast or very fast-growers or by isolates classified as slow- or very slow-growers with 54 % of all the 200 isolates belonging to fast- or very fast-growers*.* Morphologically, eighty five percent of the colonies formed on yeast extract mannitol agar were wet and gummy while 70 % were acid tolerant, i.e. they were able to grow at a pH of 3.5. Combined restriction of the 16S rRNA genes of the 60 rhizobial isolates with five restriction enzymes clearly distinguished seven different clusters at 80 % similarity level. The majority of *A. lebbeck* isolates were distinct from those of the *Acacias* and *L. leucocephala*. The *M. thonningii* isolates were related to *L. leucocephala* isolates. Simple PCR of the ITS DNA provided several distinct band sizes indicating great variation among the isolates and restriction of the ITS with three different enzymes did not yield many further differences. Molecular techniques revealed a great diversity among the rhizobia that nodulate tree legumes in the tropics and this may explain why many introduced and/or indigenous trees are able to form nodules with indigenous rhizobia in this region.

## Introduction

Rhizobia are usually defined as nitrogen-fixing soil bacteria capable of inducing the formation of root or stem nodules on leguminous plants in which atmospheric nitrogen is reduced to ammonia for the benefit of the plant. Although the majority of legumes form symbiosis with members of genera that belong to the class Alphaproteobacteria (*Allorhizobium*, *Azorhizobium*, *Blastobacter*, *Bradyrhizobium*, *Devosia*, *Ensifer*, *Mesorhizobium*, *Methylobacterium*, *Rhizobium and Sinorhizobium*), some legumes, such as those in the large genus *Mimosa*, are nodulated predominately by members of the class Betaproteobacteria in the genera *Burkholderia* and *Cupriavidus* (Gyaneshwar et al. 2011). However, a recent report by Bontemps et al. (2015) indicated that the endemic Mexican mimosas unlike their Brazillian counterparts were nodulated predominantly by Alphaproteobacteria from the genera *Rhizobium* and *Ensifer*.

In the last few years, many studies investigating rhizobia isolated from tree legumes in Kenya and Sudan have revealed considerable phenotypic and genotypic diversity among strains, and several distinct groups have been identified and novel species described (Zhang et al. 1991; Odee et al. 1997, 2002; Nick et al. 1999; McInroy et al. 1999). These studies concluded that there is a large heterogeneity among the strains (Crow et al. 1981; De Lajudie et al. 1994; Dupuy et al. 1994; Jarvis 1983; Moreira et al. 1998; Zhang et al. 1991). This indicates that trees can form nodules and fix nitrogen with several different groups of rhizobia (Crow et al. 1981; Jarvis 1983; Lindstrom et al. 1983; Padmonabhan et al. 1990).

Of late, the assessment of diversity within rhizobial natural populations in various regions of the world has received increasing attention (Amann et al. 1995; Batzli et al. 1992; Brunel et al. 1996; Cartwright et al. 1995). Many attempts have been made to determine the actual composition and characteristics of indigenous strains isolated from different cultivated legumes (Chen et al. 1991; Crow et al. 1981) and also from less explored plants (Felsenstein 1985). The development of molecular genetic methods and the availability of sensitive and accurate PCR- based fingerprinting methods (Galtier et al. 1996; Gibson 1980; Gurtler 1993) has enabled the differentiation of closely related bacterial strains and the detection of high rhizobial diversity (Gurtler and Stanisich 1996; Jarvis 1983; Jarvis et al. 1982).

In Ghana, with the exception of studies on cowpea rhizobia (Fening 1999) there is little knowledge of the diversity of rhizobia that nodulate other legumes especially tree legumes. The aim of the present study was to assess the phenotypic and genotypic characteristics of the rhizobia including their diversity that nodulate some indigenous and introduced tree legumes in Ghana.

## Materials and methods

### Soil and site characteristics

The three soil types used for the studies were taken from the Accra plains (05^o^ 39.627^1^ N, 0011.619^1`^ W). The soils belong to the Toje, Hatso and Alajo series (Local Names) (Brammer 1967). The Toje series is classified as Rhodic lixiso, and the Hatso and Alajo series classified as Haplic lixisol and Calcic vertisol, respectively, according to FAO (2006). The three soils are widely cultivated by resource-poor farmers in the area and occur on the same soil catena with Toje series being at the top, Hatso and Alajo series being at the middle and the bottom slope, respectively.

### Soil analysis

Soil pH was determined in distilled water at a soil: solution ratio of 1:1 using Pracitronic M.V 88 pH electrometer (Peech 1965). Organic carbon was determined using the wet combustion method of Walkley and Black (1934) while the Bray and Kurtz (1945) method was used to measure available phosphorus. Total nitrogen was determined by the distillation and titration method of Bremner (1965). The cation exchange capacity (CEC) of the soils was determined by extraction of the exchangeable bases using neutral ammonium acetate (NH4OAc, pH 7.0) and an aliquot was used to determined Ca, Mg, K and Na. Soil texture was determined based on particle size analysis using the modified Bouyoucos hydrometer method as describe by Day (1965).

### Initial nodulation studies

Seeds of one indigenous (*Milletia thonningii*) and four introduced (*Albizia lebbeck*, *Acacia auriculiformis*, *Acacia mangium and Leucaena leucocephala*) tree legumes were initially assessed for their nodulation potential in three different soil types by growing them in pots filled with two kilograms of each soil type for 12 weeks. Representative nodules were collected for rhizobia isolation.

### Rhizobia enumeration in the three soils

The population of indigenous rhizobia in the three different soils capable of nodulating the selected tree legumes (*Albizia lebbeck*, *Acacia auriculiformis*, *Acacia mangium*, *Leucaena leucocephala and Milletia thonningii*) were enumerated based on the most probable number (MPN) method (Vincent 1970) using plastic growth pouches (Weaver and Frederick 1972). Clean seeds of the tree legumes were surface sterilized in 70 % alcohol for 3 min and rinsed thoroughly in several changes of distilled water (Somasegaran and Hoben 1994). The seeds were scarified and pre-germinated on 1 % water agar until the radicles were about 2 cm long. Seedlings were planted two per pouch. Ten-fold dilutions of each soil sample with four replicates per dilution were used to inoculate the pouches containing N-free nutrient solution (Somasegaran and Hoben 1994). One milliliters of soil inoculants was used to inoculate each pouch. The pouches were randomly arranged in wooden racks and kept at the green house. The plants were supplied when necessary with enough N-free nutrient solution to prevent wilting and other nutrient deficiencies. The plants were assessed for nodules after 12 weeks and the most probable number of rhizobia calculated (Vincent 1970).

### Rhizobia isolation

Representative nodule samples were taken from the initial nodulation study. The nodules were surface sterilized with 70 % alcohol for 3 min and then with 0.1 % mercuric chloride for another 3 min and rinsed with several washes in sterile distilled water (Somasegaran and Hoben 1994). The nodules were each crushed in a drop of sterile distilled water in a petri dish with a sterile rod. A loop-full of the suspension was then streaked on yeast extract mannitol (YEM) agar plates and incubated at 28 °C. A total of 400 rhizobia isolates were obtained from the five legumes.

### Authentication of isolates

All the rhizobia isolates were evaluated as pure cultures that could form nodules on their respective host plants. Seeds of the leguminous plants were pre-germinated in petri-dish after scarification with conc. H_2_SO4. The pre-germinated seeds were planted in growth pouches containing N-free nutrient solution (Somasegaran and Hoben 1994). Seven days after planting, the growth pouches were inoculated with 1 ml YEM broth culture of each isolate with each treatment replicated four times. Uninoculated pouches served as control. The pouches were placed in racks and kept in the green house. Plants were harvested 12 weeks after planting and their roots assessed for the presence of nodules. Two hundred out of an initial total of 400 rhizobial isolates made up from 40 isolates each from the following five tree legume, *A. lebbeck*, *A. auriculiformis*, *A. mangium*, *L. leucocephala* and *M. thonningii*, were randomly selected for characterization and further studies.

### Culture maintenance

All rhizobial isolates were maintained on YEM slopes and stored at 4 °C. All rhizobial isolates were re-plated on YEM and checked for contamination at least every three months.

### Physiologic and metabolic characterization of the rhizobia isolates

Two hundred indigenous tree legume rhizobia isolates from the three soils were selected and used for these studies. The studies include the following:

### Growth rate, reaction to BTB and colony morphology

The time taken for the isolates to form colonies on YEM agar plates was followed for 7 days, and the ability of the isolates to change the pH of their growth medium was scored on YEM agar plates supplemented with 0.25 mg/L bromothymol blue(BTB). Plates were incubated for 7 days at 30 °C and scored daily for change in colour. Freshly prepared YEM plates containing BTB have a pH of 6.8 and are green in colour. The isolates that changed the colour of the medium to yellow were scored as acid producers and classified as fast growers. Isolates that changed the medium to blue were considered alkalisers and classified as slow growers. Colony appearance was scored as dry where the surface was smooth and firm, and wet for those which were watery or slimy.

### Growth of the tree legume rhizobia in different pH medium

The pH values of tubes containing 20 ml of YEM broth without KH_2_PO_4_ (Zablotowicz and Focht 1981), were adjusted (pH values see below) before sterilization by the addition of HCL or NaOH. The tubes were inoculated with 100 μL aliquots of each isolate and incubated at 28 °C for 7 days before scoring for growth by observation for turbidity and confirmation by colony counting on plates. Growth was determined at pH 3.5, 4.5, 5.5, 6.5 in the acid range and pH 7.5, 8.5 and 9.5 in the alkaline range.

### Ability of tree legume rhizobia to utilize different carbon

The rhizobia isolates were tested for their ability to grow when provided with different carbohydrates as the sole carbon source. The test was carried out in a standard basal medium without mannitol (Zablotowicz and Focht 1981). Each carbohydrate was added to a final concentration of 10 % (*W*/*V*). The carbohydrates were sterilized by filtration through Millipore membranes (pore size 0.22 μm) and then added to the sterilized liquefied medium just before plates were poured. Each isolate was analysed on duplicate plates and the growth scored after incubation for seven days at a temperature of 28 °C. The following carbohydrate sources were tested: L-arabinose, D-glucose, D-galactose, fructose, lactose, maltose, mannitol and sucrose.

### Molecular characterization of some tree legume rhizobial isolates

A total of 60 tree legume rhizobial isolates which were considered as representative strains of the 200 isolates were selected for further molecular studies.

### DNA extraction.

The rhizobial isolates were cultivated on YEM agar plates at 28 °C for 5 days. Single colonies of the rhizobial isolates were picked, washed in 100 μl TE at _P_H 7.5 and spun down (Sally et al. 2010). 250 μl of CTAB buffer were added to the washed cells; they were vortexed for 30 s, and incubated at 65 °C for 15 min and then cooled down to room temperature (Sally et al. 2010). 250 μl of 24:1 chloroform: isoamyl alcohol was added to the samples and vortexed thoroughly. The suspension was then centrifuged for 10 min at 12,000 rpm using a fixed angle rotor. The aqueous phase was transferred to a new sterile 1.5 ml micro centrifuge tube and equal amounts of cold isopropanol added and mixed gently (Sally et al. 2010). The DNA was precipitated at -20 °C for 30 min and centrifuged for 10 min at 12,000 rpm. The DNA was re-suspended in 30 μl TE buffer at _P_H 7.4 and the concentration of the extracted DNA assessed at 260 nm using a Nanodrop Spectrophotometer (Sally et al. 2010).

### PCR amplication of 16S rRNA gene

The universal primers fD1 (5^1^- AGAGTTTGATCCTGGCTCAG-3^1^) and rD1. (5^1^- AAGGAGGTGATCCAGCC-3^1^) were used for PCR amplification of the 16S rRNA (Weisburg et al. 1991). fD1 and rD1 are primers derived from conserved regions of the 16S rRNA genes and amplify nearly full length of 16S rRNA genes (Weisburg et al. 1991). Amplification reactions were performed in a total volume of 25 μl and contain the following: 1× reaction buffer (10 Mm Tris-Hcl, 50 MmKcl) with 1.5 mM MgCl_2_, 2.5 units Taq polymerase, 200 μM of each dNTP (dATP, dCTP, dGTP and dTTP ), 5 pmol of each forward and reverse primer and 100 ng of genomic DNA. The temperature profile was as follows: Initial denaturation at 95 °C for 3 min; 35 cycles of denaturation at 94 °C for 1 min, annealing at 55 °C for 1 min, extension at 72 °C for 2 min and final extension at 72 °C for 3 min. The amplified products were kept at a temperature of 4 °C. All amplifications were carried out in Thermocycler (Bio-Rad).

### PCR amplification of 16S-23S intergenic spacer (ITS)

The intergenic region between the 16S and the 23S rRNAs was amplified by PCR with primers derived from the 3^1^ end of the 16S rRNA (FGPS1490-72; 5^1^-TGCGGCTGGATCCCCTCCTT-3^1^) and from the 5^1^ end of the 23S rRNA (FGPL132-38; 5^1^-CCGGGTTTCCCCATTCGG-3^1^) (Ponsonnet and Nesme 1994). The amplification reactions and conditions were the same as those used for 16S rRNA amplification.

### Gel electrophoresis and imaging

The resulting amplicons (5 μl) of the 16S rRNA and ITS genes were mixed with loading buffer (2 μl) and analysed electrophoretically through 1 % agarose gel stained with ethidium bromide (10 mg/ml) at 100 V for 1 h. The bands were observed and photographed under UV light using BioDoc-IT imaging system.

### Restriction fragment length polymorphism (RFLP) analysis of the 16S rRNA and ITS

Aliquots (10 μL) of the 16S rRNA and ITS PCR products were digested with restriction endonucleases as specified by the manufacturer (Bioron GmbH) with 5 U of enzyme per reaction in a total volume of 25 μL. The following enzymes were used for the 16S amplicon digestion: HaeIII, AluI, HPaII, HPaI and RsaI. The ITS amplicons were digested with three enzymes namely HaeIII, Alu I and HPaII.

The restriction fragments were separated by horizontal electrophoresis in TBE buffer (89 mM Tris, 89 mM boric acid, 2 mM EDTA (pH 8.0) on a 2 % (*w*/*v*) agarose gel containing 10 mg of ethidium bromide per ml. The gels were run at 80 V for 2 h and immediately photographed under UV light using Bio-Doc-IT imaging system.

## Results

### Soil properties

The characteristics of the soils used in this study are presented in Table 1. The Alajo series with a clay loam texture had the highest values for all parameters measured, while the Hatso series, a sandy soil was lowest in total N, organic carbon and available P, whereas the Toje series, a sandy clay loam was lowest in pH and CEC values.

**Table 1:** Chemical properties of the soils used for this study

Parameters	Soil types		
	Toje	Hatso	Alajo
pH	5.3	6.0	6.8
Total N (g kg ^−1^)	0.59	0.34	1.34
Organic carbon (g kg ^−1^)	6.5	3.7	13.9
CEC (cmol kg ^−1^)	5.84	7.40	25.20
Available P (mg kg ^−1^)	7.52	3.76	12.4
Texture	Sandy clay loam	Sandy	Clay loam

### Enumeration of native rhizobia capable of nodulating five selected indigenous tree legumes

Table 2 provides information on the population densities of native rhizobia capable of nodulating five of the tree legumes in the three soil types. The results indicate that, native rhizobia capable of nodulating each of the five tree legumes are present in all the three soils used for the study with the native rhizobia population ranging from 22 per gram of soil in Alajo soil to 5200 per gram of soil in either Hatso or Toje soil. In all cases, the highest rhizobia population per gram of soil capable of nodulating the tree legumes was highest in Hatso soil, followed by Toje soil and lowest in Alajo soil.

**Table 2:** Indigenous rhizobia population /g soil in the Toje, Hatso and Alajo soils

Tree species	Rhizobia population /g of soil		
	TOJE	HATSO	ALAJO
*Albizia lebbeck*	780	5200	1800
*A. auriculiformis*	520	1800	180
*A. mangium*	180	210	22
*Leucaena leucocephala*	780	1800	650
*Milletia thonningii*	5200	5200	780

### Physiologic and metabolic properties of some indigenous tree legume rhizobia

The rhizobia that nodulated the five tree species under analysis were diverse in colony morphology, reaction with BTB, growth at different pH ranges as well as in their ability to utilize different carbon sources.

**Fig 1:**
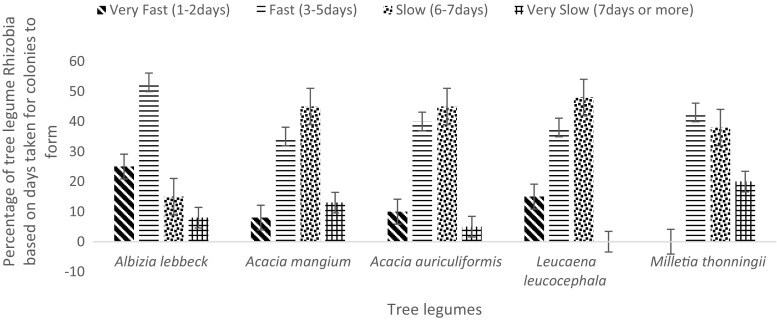
Classification of 40 rhizobial isolates obtained from each of the five tree legumes based on number of days taken for colonies to form on YEM

Fifty four percent (54 %) of the total isolates acidified YEM medium and were considered as fast or very fast growers with the rest (46 %) alkalizing the medium and classified as being slow or very slow growers. Each tree legume harboured all the various types of rhizobia strains in its nodules (Fig. 1). While both types were represented in the nodules on each tree, which one was in the majority was dictated by the tree species (Fig. 1). Thus while *A. mangium*, *A. auriculiformis* and *L. leucocephala* were predominantly nodulated by slow-growers strains with 45 %, 45 % and 48 % respectively, the other tree legumes; *A. lebbeck* and *M thonningii* were predominantly nodulated by fast-growers (Fig. 1). For example, 53 % and 43 %, respectively, of the rhizobia that nodulated *A. lebbeck* and *M. thonningii* were fast growers.

**Fig. 2:**
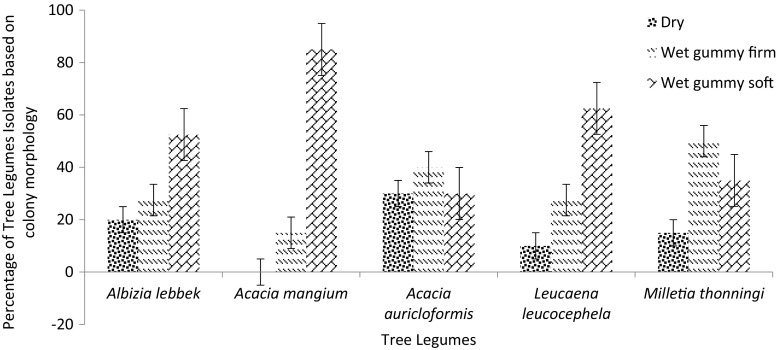
Classification based on colony morphology of 40 rhizobial isolates that nodulated each of the five tree legumes

Each tree legume was nodulated by rhizobia forming all three colony types, dry (D), wet gummy firm (WGF), and wet gummy soft (WGS) except for *A. mangium* that was nodulated by only wet gummy firm and wet gummy soft rhizobial isolates (Fig. 2). Apart from *A. auriculiformis* and *M. thonningii* which were predominantly nodulated by wet gummy firm rhizobia isolates, all the other tree legumes were predominantly nodulated by wet gummy soft rhizobial isolates (Fig. 2). For instance, *A. lebbeck*, *L. leucocephala* and *A. mangium* were predominantly nodulated by 50 %, 62.5 % and 80 %, respectively, of wet gummy soft rhizobia isolates. In all, only 15 % of the total isolates were considered dry whilst 32.5 % and 52.5 % were considered wet gummy firm and wet gummy soft, respectively. All the isolates from the Alajo soil series that nodulated the tree legumes were considered wet gummy soft but the Toje and Hatso soil series harboured all the three types of isolates (i.e., dry, wet gummy firm and wet gummy soft).

**Fig. 3:**
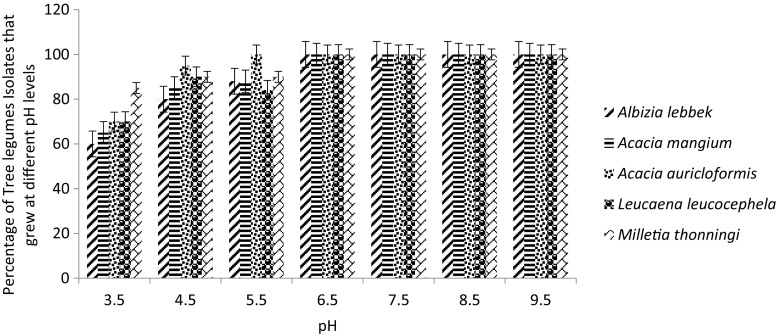
Effect of six different pH levels on growth of 40 rhizobial isolated from each of the five tree legumes

Isolates from each tree legume varied in terms of their ability to grow in media at different pH levels (Fig. 3). In general, the inability of the tree legume rhizobial isolates to grow in media with different pH increased with decreasing pH levels. For instance, at a pH of 3.5, 40 % and 35 %, respectively, of *A. lebbeck* and *A. mangium* rhizobial isolates did not grow and also 30 % each of *A. auriculiformis* and *L. leucocephala* rhizobial isolates could not grow. In contrast, almost all the tree legume isolates could grow in the alkaline pH range from 7.5 to 9.5 (Fig. 3).

Thirty percent of the total of 200 isolates could not grow at a pH of 3.5 while 13.5 % and 7 %, respectively, could not grow at pH 4.5 and 5.5, respectively. The majority of the total isolates that could not grow at acidic pH ranges (pH 3.5, 4.5 and 5.5) were fast growers*.* However, more than 77 % and 71 %, respectively, of the total isolates that could not grow at pH 4.5 and 5.5, respectively, were slow growers.

### Molecular characterization of 60 rhizobial isolates of some tree legumes

Sixty of the rhizobia isolates were randomly selected as representative strains of the tree legumes rhizobia after phenotypic characterization. The relationships among the isolates were then assessed using molecular tools. Table 3 provides a list of strains used for this study and their host legumes.

**Table 3:** List of isolates used in this studyand their respective host legumes

Host Tree Legumes	Isolate numbers
*Albizia lebbeck*	2, 3, 4, 6, 7, 8, 9, 10, 15, 30, 42, 45, 48, 51.
*Acacia mangium*	5, 11, 12, 13, 14, 17, 20, 53, 58, 61, 62.
*Acacia auriculiformis*	1, 16, 21, 23, 24, 25, 26, 28, 52, 54, 55, 56, 57.
*Leucaena leucocephala*	19, 22, 27, 31, 33, 35, 37, 38, 39, 40, 50
*Milletia thonningii*	18, 29, 32, 34, 36, 43, 44, 47, 49, 59, 60

### PCR amplification and RFLP analysis of 16S rRNA genes of some tree legumes rhizobia

**Fig. 4:**
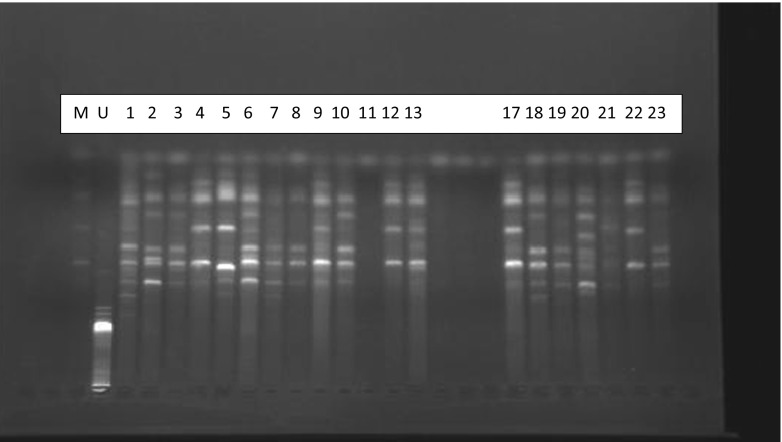
Restriction patterns of PCR-amplified 16S rRNA digested with HpaII. Molecular size marker (M): 2 k bp ladder. U is the control representing an uncut 16S rRNA amplicon

PCR amplification of 16S rRNA genes of almost all the 60 rhizobia isolates of the tree legumes produced a single band 1.5 kb in size. Restriction of the 16S rRNA amplicons of the rhizobial isolated from the different legumes with any of the restriction enzymes Hae111, Alu1, HpaI, HpaII and Rsa1 produced multiple bands. Although each restriction enzyme produced polymorphic band patterns, the most diverse were those obtained with Rsa1 and HpaII; the latter are shown in Fig. 4.

**Fig. 5:**
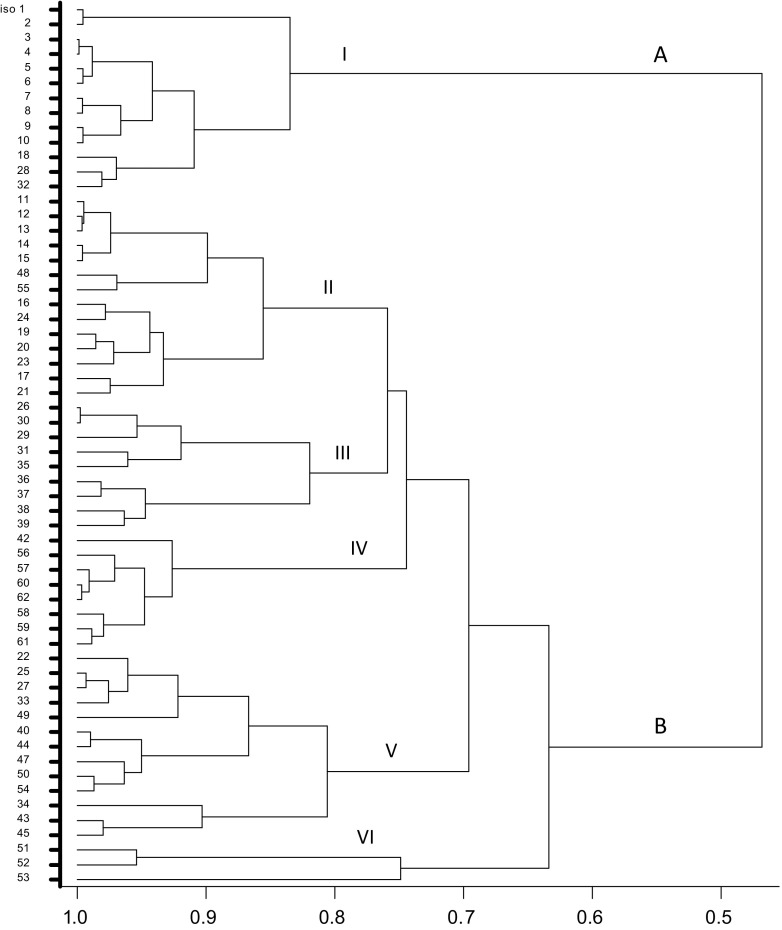
Dendrogram of indigenous tree legume rhizobia based on combined HaeIII, RsaI, HpaI, HpaII and AluI restriction patterns of amplified 16S rRNA. The data were analysed using UPGMA in the BioNumerics program

Combined restriction of the 16 S rRNA amplicons with the five restriction enzymes distinguished clearly between two major clusters A and B at 45 % similarity levels which were further separated into six distinct clusters (I - VI) at 80 % similarity level (Fig. 5). Out of a total of sixty isolates, cluster A consisted of 13 isolates that were genetically distinct from cluster B which consisted of 47 isolates. None of the clusters (I-VI) contained isolates obtained from all the five tree legumes. Rather, each cluster contained isolates that were dominated by rhizobia from one or two legume species. For instance, most of the isolates in cluster I were those obtained from *A. lebbeck* (62 %), while cluster II was dominated by isolates obtained from the two acacias (79 %). Almost 56 % of the isolates in cluster III were from *L. leucocephala.* Isolates obtained from *M. thonningii* and *L. leucocephala* which appeared similar at 80 % similarity level also formed almost 77 % of the isolates in cluster V. Isolate 53 obtained from *A. mangium* appeared distinct at 80 % similarity level. Generally, the majority of the isolates were separated at a similarity levels between 80 % and 90 %.

### PCR amplification and RFLP analysis of the 16S and 23S rRNA intergenic spacer (ITS) amplicon of some isolates from indigenous tree legumes

**Fig. 6:**
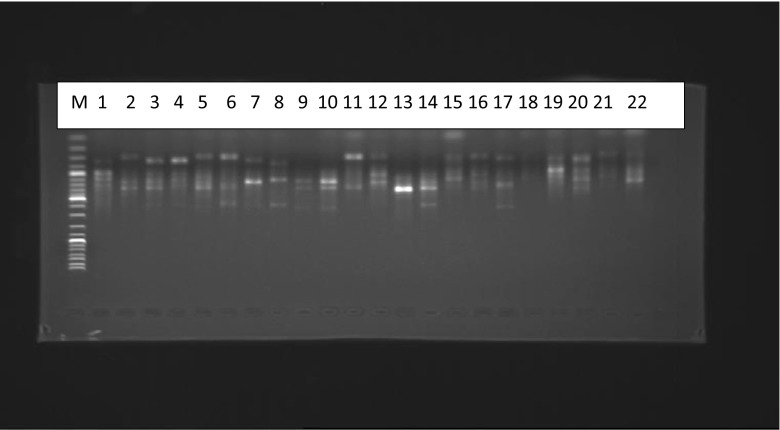
Patterns obtained with undigested ITS amplicons of some tree legume isolates

PCR amplification of the intergenic spacer (ITS) between the 16S and 23S rRNA genes of all the strains produced multiple polymorphic bands with size ranging from 800 bp-1.4kbp (Fig. 6). Cluster analysis of undigested PCR product of the isolates ITS shows that, the isolates could be grouped into two main clusters namely A and B at about 10 % similarity level (Fig. 5). Cluster A consist of isolates that were predominantly obtained from *A. lebbeck*, *A. mangium* and *A. auriculiformis* whiles cluster B consist of isolates that were predominantly obtained from *L. leucocephala* and *M. thonningii*. Each cluster was further separated into sub-groups with each sub-group containing a mixture of isolates obtained from all the tree legumes.

**Fig. 7:**
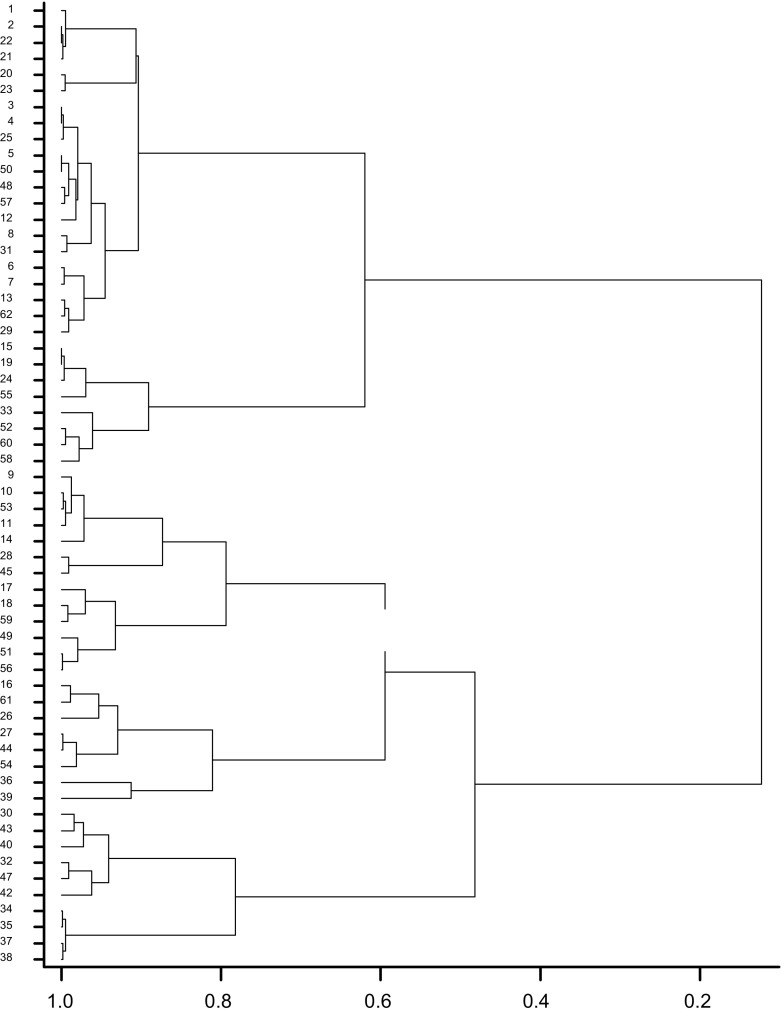
Dendrogram of some indigenous tree legume rhizobia isolates based on undigested ITS patterns. The data were clustered by using UPGMA in the BioNumerics program

Restriction of the ITS with the three enzymes HaeIII, AluI and HpaII did not allow much further differentiation among the isolates.

## Discussion

Bacteria belonging to the family of *Rhizobiaceae* were divided into two groups on the basis of their growth rate on YEM medium. These were the fast-growing rhizobia and the slow-growing rhizobia. The results showed that each tree legume was nodulated by both types of rhizobia, but which one formed the majority was dependent on the tree species. The results also showed presence of both types of rhizobia in all the three soils studied, which is consistent with their appearance in many tropical soils as reported in earlier studies (Sanginga et al. 1989; Dreyfus and Dommergues 1981).

This has also been reported by other researchers (Moreira et al. 1998; Trinick 1980; Turk and Keyser 1992; Zhang et al. 1991).

Some studies (e.g. Sanginga et al. 1989) gave the impression that slow-growing rhizobia dominate in tropical soils, Our results showed the reverse with 54 % of nodules of all five trees being formed by fast- growing species. Sanginga et al. (1989) reported that although *L. leucocephala* was nodulated by both fast and slow-growing rhizobia, only effective nitrogen fixing nodules were formed with the fast-growing rhizobia. These findings suggest that the fast-growing rhizobia are more important in N_2_ fixation in tropical soils than has been assumed.

On the basis of rhizobial growth on YEM medium amended with a pH indicator, the isolates were classified as acid producers or as alkalizers. Our results showed that each legume was nodulated by both types of rhizobia although the acid producers were more prominent than the alkalizers. This suggests that acid producers are important in nodulation and nitrogen fixation in tree legumes in tropical soils which tend to be acidic or slightly acidic.

The high rhizobial population observed in the three soils might be due to the fact that these soils had earlier been used to cultivate legumes belonging to the same cross inoculation groups as the tree legumes. Fast-growing rhizobia are generally considered to be less tolerant of acidic conditions than slow-growing strains (Graham et al. 1994), However, analysis of the pH tolerance of the tree legume rhizobia indicated that most of the tree legume rhizobia were fast-growers and acid tolerant. Indeed 70 % of all isolates could grow at a pH of 3.5. Other researchers have found the existence of fast-growers that are tolerant to acid conditions (Cooper et al. 1985; Cunningham and Munns 1984, Graham et al. 1994, Wood et al. 1988). Further support for the existence of acid-tolerant fast-growing strains come from studies on strains nodulating *Vigna unguiculata* which are tolerant to pH values as low as 4 (Mpepereki et al. 1997.) This tolerance to acidic conditions may reflect the fact t that the majority of the strains were isolated from slightly to moderately acid soils and hence were adapted to such conditions.

Amplification of the 16S rRNA gene of almost all the rhizobia isolates used in this study resulted in a single band 1.5 kb in size. This band size correspond to the expected size reported earlier by Weisburg et al. (1991) and Terefework et al. (1998). Combined restriction of the 16S rRNA genes of the rhizobia isolates with five endonucleases distinguished clearly six different combinations of patterns or fingerprints at 80 % similarity level which represents six distinct 16S rRNA genotypes among the isolates. This finding indicates great variations among the isolates and suggests that the soils harbour populations of highly diverse strains that nodulate the five tree legumes species. This finding is in agreement with the results obtained in other parts of the world (Ando and Yokoyama 1999; Niemann et al. 1997).

Characterization of the rhizobial isolates based on PCR of the ITS sequence provided several distinct band size indicating great variation among the isolates. The high level of ITS size heterogeneity is consistent with the findings by Zerhari et al. (1998), who performed an extensive study of the phenotypic and genotypic characteristics of rhizobia nodulating *Acacia spp*. in Morocco and concluded that these rhizobia seem to belong to several different clusters. Such length variability of the ITS was also recorded by Laguerre et al. (1996), between genotypes within *R. leguminosarum*. However, further restriction of the ITS with three restriction enzymes did not result in any additional distinction among the strains examined. This finding is similar to results obtained by Khbaya et al. (1998), who investigated genetic diversity and phylogeny of several rhizobial strains that nodulate *Acacia* species using PCR with RFLP. Lack of major difference in the restriction patterns of the rhizobia ITS relative to their unrestricted sizes suggests that there is little sequence variability in the ITS among the tree nodulating rhizobia that cannot be revealed simply by RFLP analysis. The occurrence of a wide diversity of strains in any soil increases the opportunity for various legume hosts to find compactible rhizobia for nodulation. Although all the characterization methods used, phenotypic or genotypic, revealed considerable diversity in the rhizobia that nodulated the different tree species, the molecular methods were superior for identification and classification.

Many developing countries do not have facilities for inoculant production and therefore need to rely on the indigenous rhizobia to nodulate their legumes. It can be concluded that the high diversity of tree-nodulating rhizobia found in this study and by others (Crow et al. 1981; Jarvis 1983; Padmonabhan et al. 1990), provides sufficient effective stains of indigenous rhizobia to enhance growth of native and introduced trees in the often N-depleted soils of the tropics.
